# Combating bovine mastitis: current insights and future directions for a global dairy challenge

**DOI:** 10.5194/aab-69-157-2026

**Published:** 2026-03-12

**Authors:** Shah Zeb Ahmad, Wenjuan Zhao, Ye Feng, Xubin Lu, Fagang Zhong, Mengli Han, Rifat Ullah Jan, Muhammad Irfan Khan, Zhangping Yang, Zhi Chen

**Affiliations:** 1 College of Animal Science and Technology, Yangzhou University, Yangzhou 225009, China; 2 State Key Laboratory for Sheep Genetic Improvement and Healthy Production, Xinjiang Academy of Agricultural and Reclamation Science, Shihezi 832000, China

## Abstract

Bovine mastitis is a costly inflammatory condition of the mammary gland that continues to pose a significant threat to the dairy industry and food safety worldwide. This review summarizes our understanding of mastitis; its classification into clinical and subclinical forms; and the predominant bacterial pathogens that cause bovine mastitis, namely *Staphylococcus* species. The effects of mastitis on milk yield and composition, as well as its significant economic impact, are also examined. Emerging diagnostic biomarkers and the role of melatonin in regulating immune and antioxidant responses during infection are also included in this review. A critical review of control measures highlights the limitations of traditional antibiotic treatments due to increasing antimicrobial resistance (AMR). We investigate alternative methods, including improved herd management, nutrition, vaccinations, essential oil therapy, bacteriophage therapy, and nanotechnology. Finally, the research gaps identified in this review include genetic host susceptibility, the roles of the mammary and gut microbiomes, and the need for standardized clinical trials of novel therapies. This review seeks to assist in identifying a plan for expanded and new research on integrated, environmentally sustainable solutions for the prevention and control of mastitis.

## 1 Introduction

Mastitis is among the most significant and serious production diseases in dairy cattle and is now considered a serious threat to dairy farming due to its consequences, ranging from animal health to substantial economic losses (Zigo et al., 2021; Trinidad et al., 1990). It can be characterized by chemical and bacteriological variations in the animal product – that is, milk. Other diagnostic methods include pathological changes in the mammary gland, which are the consequences of host cell immune responses aimed at eliminating inflammation-triggering pathogens (Hodges et al., 1984). Recently, other biomarkers have also been identified for the diagnosis of mastitis (Carvalho-Sombra et al., 2021; Huma et al., 2020; Antanaitis et al., 2021). In addition to bacterial mastitis, many other inflammatory agents, such as exposure to chemical irritants, physical trauma, and other pathogens and their toxins, cause mammary gland inflammation (Hodges et al., 1984). Regarding susceptibility, it is now clear that crossbred cattle are more prone to infection than purebred cattle, particularly in calves, which may serve as vectors for the spread of mastitis (Kibebew, 2017).

Globally, dairy products, especially milk, are consumed because of their nutritional value (Strydom et al., 2025). The global population is increasing and is projected to reach 9.7 billion in 2050, as reported by the United Nations Department of Economic and Social Affairs (UN DESA), Population Division (Didanna and Anja, 2025), which would also increase demand for dairy products. An increase in dairy production is expected, with dairy product consumption reaching 15.2 L per capita (FAO, 2023). Hence, mastitis poses a risk to public health by reducing milk production and altering milk composition, thereby lowering milk quality standards, with an estimated loss of EUR 185 per cow (Dubuc et al., 2011). One survey-based study found that ∼ 23 % of cows were induced to calve too early due to udder health concerns (Turk et al., 2017). Apart from livestock, it is a common infection in humans, resulting in premature weaning and stunted infant growth (Ruegg, 2017; Aryeetey et al., 2008; Seegers et al., 2003; Hillerton and Berry, 2005). In mastitis-affected dairy cattle, milk production drops by 30 % in each quadrant, resulting in a 15 % loss per milking cow per lactation (Abutarbush, 2010). The somatic cell count (SCC) is also associated with pathogenic strains, such as *Enterococcus* species, which significantly affect SCC (Joy et al., 2023). Based on the current status and importance of mastitis, this review summarizes the literature on the relationship between mastitis and the expression of various genes, the secretion of melatonin, and their role in reducing oxidative stress, as well as treatment strategies used to overcome the infection, consequently safeguarding animal health and economic losses to the dairy industry. In addition, we have identified some research gaps in the aforementioned research areas that could be fruitful for researchers and aid existing knowledge about mastitis.

## 2 Methodology

### 2.1 Databases and search strategy

This narrative review was conducted by searching databases, including PubMed, Web of Science, Google Scholar, and Scopus, using relevant keywords, such as “bovine mastitis” and “dairy cows”, to identify studies on the etiology, occurrence, prevention, and treatment of bovine mastitis.

### 2.2 Eligibility criteria and study selection

Articles published in the last two decades (i.e., from 2005 to 2025) were extensively studied. More focus was given to articles from the last decade; however, outdated studies were also included when it was not possible to obtain more recent articles. The studies included original and review articles that specifically focus on bovine mastitis.

## 3 Causes of mastitis and their classification

Animal biologists classified the causes of mastitis primarily into two fundamental groups. The well-known group is, in fact, infection caused by microbial pathogens. Second, mastitis is caused by external factors like practicing incorrect technological procedures in milking, udder injuries, metabolic disorders, and other stress-inducing factors (Holko et al., 2019).

Approximately 137 organisms have been described as causing bovine mastitis, including bacteria, yeasts, viruses, algae, and mycoplasmas (Holko et al., 2019). Among these pathogens, bacteria are the main and prominent causative agents of bovine mastitis, accounting for 95 % of all cases (Hoque et al., 2020; Zigo et al., 2019), with *Staphylococcus aureus* and *Streptococcus *spp. among the most common (Cvetnić et al., 2016). In most cases of mastitis, only a single bacterial species is identified from the milk sample of diseased cattle; however, this does not mean that cases of mastitis with more than one pathogen simultaneously are not possible, as cases have been identified in which more than one bacterial species were identified from the milk sample of diseased mammary glands (Zigo et al., 2021).

These bacterial pathogens include *Staphylococcus aureus, Streptococcus agalactiae*, and *Escherichia coli*. These bacteria were collected from cows with subclinical mastitis (Hameed et al., 2017; Ewida and Al-Hosary, 2020). One study has also shown that rumen microbiota are associated with bovine mastitis, providing a foundation for therapeutic strategies (Hu et al., 2022; Rainard, 2017). According to Fredebeul-Krein et al. (2022), *Streptococcus uberis* and coliform bacteria are the main causative agents of moderate to severe mastitis, characterized by ketosis and uterine abnormalities in cows. Moreover, the study also showed that coliform bacteria were less prevalent than formal bacteria. Other bacteria, such as *Streptococcus dysgalactiae*, *Klebsiella oxytoca*, and *Klebsiella pneumoniae*, have been isolated from diseased cows (Abdi et al., 2021).

Mastitis caused by bacteria can be environmental or contagious. Environmental mastitis, referred to as “mastitis from the surrounding environment”, means that the microorganisms that cause mastitis come from the external environment, implying that these pathogens thrive in the environment (Zigo et al., 2021). These pathogens include *E. coli, Klebsiella pneumoniae*, *Enterobacter aerogenes*, *Streptococcus uberis* (Ozbey et al., 2024; Smith et al., 1985), *S. aureus*, and *Streptococcus agalactiae* (Klaas and Zadoks, 2018). Many other bacterial strains include *Streptococcus uberis*, coagulase-negative staphylococci (CoNS), *Corynebacterium* spp., *Pseudomonas* spp., *Serratia* spp., *Proteus* spp., *Pasteurella* spp., *Listeria* spp., *Leptospira* spp., *Yersinia* spp., *Enterobacter* spp., *Brucella* spp., and *Mycobacterium* spp. Feces are the main source of environmental mastitis, and the main route of entry is the orifice of the mammary teats, teats' ducts, which are exposed for up to 2 h after milking (Nemeth et al., 1994; Jones and Bailey, 2006), as well as through microlesions and damaged udder skin (Trinidad et al., 1990; Sol et al., 1994).

In contrast, contagious mastitis refers to the spread of pathogens between udder quarters (Zigo et al., 2020; Klaas and Zadoks, 2018; Smith et al., 1985), indicating that these pathogens can survive and proliferate inside the mammary gland (Webster, 2020). As reported by Zigo et al. (2019), the most common contagious mastitis-causing agents are *S. aureus*, *Streptococcus agalactiae*, and *Streptococcus uberis* (Table 1).

**Table 1:** Different types of mastitis-causing agents, along with their pathogenicity and prevalence in different regions.

Pathogenicity	Transmission	Bacterial strain	Type of mastitis	Prevalence in selected Asian countries	References
Major	Environmental	*Staphylococcus aureus*	Subclinical &clinical	China, Pakistan, India, Nepal, Türkiye, Bangladesh, USA, Slovakia, Ethiopia	Ewida and Al-Hosary (2020);Hameed et al. (2017);Al-Harbi et al. (2021);Cobirka et al. (2020);Bhattarai et al. (2020); Bi et al. (2016);Kirkan et al. (2005); Sahoo et al. (2023); Preethirani et al. (2015); Turkyilmaz et al. (2010); Yalcin et al. (2024); Zaman Faruk et al. (2025)
Major	Environmental	*Streptococcus* spp.	Subclinical &clinical	China, Pakistan, India, Nepal,Türkiye, Bangladesh, USA, Slovakia,Ethiopia	Ewida and Al-Hosary (2020);Hameed et al. (2017);Al-Harbi et al. (2021);Cobirka et al. (2020);Bi et al. (2016);Sahoo et al. (2023); Preethirani et al. (2015);Turkyilmaz et al. (2010); Yalcin et al. (2024); Zaman Faruk et al. (2025)
Major	Environmental	*Escherichia coli*	Subclinical &clinical	China, Pakistan, India, Nepal, Türkiye, Bangladesh, USA, Slovakia, Ethiopia	Ewida and Al-Hosary (2020);Hameed et al. (2017);Al-Harbi et al. (2021);Cobirka et al. (2020);Bi et al. (2016);Sahoo et al. (2023); Preethirani et al. (2015); Turkyilmaz et al. (2010); Yalcin et al. (2024); Zaman Faruk et al. (2025)
Major	Contagious	*Staphylococcus aureus*	Subclinical	China, Pakistan, India, Nepal, Türkiye, Bangladesh, Slovakia, USA	Ewida and Al-Hosary (2020);Hameed et al. (2017);Cobirka et al. (2020);Bi et al. (2016);Sahoo et al. (2023); Preethirani et al. (2015); Turkyilmaz et al. (2010); Yalcin et al. (2024); Zaman Faruk et al. (2025)
Minor	Environmental	*Klebsiella* spp. (*K. oxytoca*, *K. pneumoniae*)	Clinical	China, Pakistan, India, Bangladesh, USA, Slovakia	Cobirka et al. (2020);Zadoks and Fitzpatrick (2009);Bi et al. (2016);Sahoo et al. (2023); Preethirani et al. (2015); Zaman Faruk et al. (2025)
Minor	Environmental	*Corynebacterium* spp.	Clinical	China, India	Bi et al. (2016);Sahoo et al. (2023); Preethirani et al. (2015); Turkyilmaz et al. (2010); Yalcin et al. (2024)
Minor	Environmental	Other NAS^*^ (*S. chromogenes*, *S. haemolyticus*, *S. epidermidis*, etc.)	Clinical	India, Türkiye, Slovakia, Ethiopia	Bi et al. (2016);Sahoo et al. (2023); Preethirani et al. (2015); Turkyilmaz et al. (2010); Yalcin et al. (2024)
Minor	Contagious	*Streptococcus agalactiae*	Clinical	India, Türkiye, USA, Slovakia, Belgium, Germany, Brazil, Uruguay, Denmark, Norway, UK	Bi et al. (2016);Sahoo et al. (2023); Preethirani et al. (2015)

^*^ NAS = : non-aureus staphylococci. Non-aureus staphylococci are a heterogeneous group of Gram-positive, catalase-positive, and typically coagulase-negative bacteria within the *Staphylococcus* genus, excluding *Staphylococcus aureus*.

Inflammation of the mammary gland depends on the degree of reaction of the udder tissue to external forces, such as injury or infection (Tančin et al., 2013). This inflammation of the mammary gland is also linked to the interplay between cows' innate and adaptive immunity, and the type, concentration, and pathogenicity of infectious agents. For instance, infection of the mammary gland by a large number of virulent pathogenic strains results in clinical or chronic mastitis (Broucek et al., 2015; Mbindyo et al., 2020) (see Table 1).

Clinical or chronic mastitis occurs suddenly, with more profound changes in milk composition and appearance, as well as a decrease in milk quantity. The signs include inflammation in the infected udder quarter. In comparison with clinical mastitis, subclinical mastitis results from the introduction of less-virulent pathogens at lower concentrations, and the absence of observable signs and symptoms. Despite the lack of signs and symptoms, a decrease in milk production along with an increase in somatic cell count (SCC, the only diagnostic indicator) makes it a more prevalent type of mastitis, making it economically the most important and having long-term effects. Sometimes, in some cases, subclinical mastitis remains throughout the period of lactation in cows without any profound clinical signs. In some cases, observable symptoms occur in repeated episodes, also called mild clinical mastitis. In this stage, cows are considered at risk of cross-infection due to continuous shedding of pathogens (Sharma and Jeong, 2013).

The most prominent cause of mastitis is bacterial strains of the genus *Staphylococcus*, i.e., most infected cows harbor *Staphylococcus* (coagulase-negative staphylococci (CoNS), 32 %, and *S. aureus*, 11 %) (Al-Harbi et al., 2021; Abdi et al., 2021). *S. aureus* is found on the skin of cows and humans without causing any harm, even after it penetrates the cow's teat due to some external factors, like injury. Whenever these bacteria enter the teats or teat ducts (in sufficient numbers), the cow develops one of the clinical forms of mastitis. Signs of clinical mastitis include an inflamed, infected quarter that is extremely painful, due to which the cow becomes sessile and cannot move easily. The infected mammary gland quarter secretes blood-stained serous fluid. On the other hand, CoNS bacteria cause minor infections in cows, but scientists are still working to develop treatments for mastitis caused by CoNS strains. The fact is that these bacteria are adapted to resist the antibiotics commonly used. They form a biofilm around themselves, thereby resisting antibiotics (Pyörälä and Taponen, 2009; Otto, 2004; Moniri et al., 2007; Melchior et al., 2006). However, CoNS is less virulent than *S. aureus*. Still, it has been reported that CoNS produces enterotoxins (dos Santos Nascimento et al., 2005), which make this bacterial strain virulent and are responsible for the development of clinical mastitis (Haveri et al., 2007; Vasiľ et al., 2012). A rare type of mastitis called peracute mastitis sometimes occurs during early lactation, when the cows' immune response is depressed. In peracute mastitis, the cows become seriously ill, with high fever, loss of appetite, and depression. The animals may become comatose in certain cases and die in approximately 24 h after the onset of symptoms (Webster, 2020).

Based on prevalence and pathogenicity, mastitis-causing pathogens are divided into two categories: major and minor. The major agents include *E. coli, S. aureus*, and streptococci. These major pathogens are more harmful because of their zoonotic nature, which allows them to be easily transferred to humans (Zi et al., 2018). At the same time, the minor agents include *S. chromogenes*, *S. haemolyticus*, *S. epidermidis*, *S. simulans*, *S. sciuri*, and *Corynebacterium bovis* (Cobirka et al., 2020). Table 1 summarizes the major and minor mastitis-causing agents (Table 1).

Apart from bacterial inflammation, many studies have identified residual chemicals involved in mammary cell inflammation. These chemicals include difenoconazole (DIF), a triazole fungicide in the azole class. It is one of the chemicals that has gained importance in agriculture for controlling various plant pathogens (Rather et al., 2022; Marichal et al., 1990). Na et al. (2024) found that difenoconazole induces inflammation in mammary epithelial cells via endoplasmic reticulum stress and inflammatory response. Furthermore, the fungicide tetraconazole can impair milk production after being applied to bovine mammary epithelial cells by disrupting calcium homeostasis and mitochondrial function (Jeong et al., 2023). Bifenox, a herbicide, has also been identified as an ER stressor and a cell-inflammation-causing agent, leading to the death of bovine mammary epithelial cells and decreased casein levels (You et al., 2023). Another synthetic pyrethroid insecticide, bifenthrin, is now regarded as a mitochondrial membrane potential (MMP) reducer, thereby disrupting cell proliferation by increasing reactive oxygen species (ROS) (Sung et al., 2023). Despite their importance in pest management, these chemicals pose serious risks to cattle and humans, which botanists should consider when developing pest management systems.

## 4 Secretion of melatonin during mastitis infection

During systemic stress induced by mastitis, activation of the hypothalamic-pituitary-adrenocortical (HPA) axis and the sympathetic nervous system (SNS) releases norepinephrine (NE), which primarily suppresses antiviral type-1 interferons (IFN-1). NE also regulates transcription of genes associated with the proinflammatory immune response, i.e., the synthesis of IL-1, THF, and IL-6. IL-1 β is an inflammatory cytokine that acts as a pyrogen by upregulating prostaglandin secretion, neutrophil activation, T and B lymphocyte activation, and antibody production. It also helps in fibroblast and collagen production (Zefferino et al., 2021).

**Figure 1:**
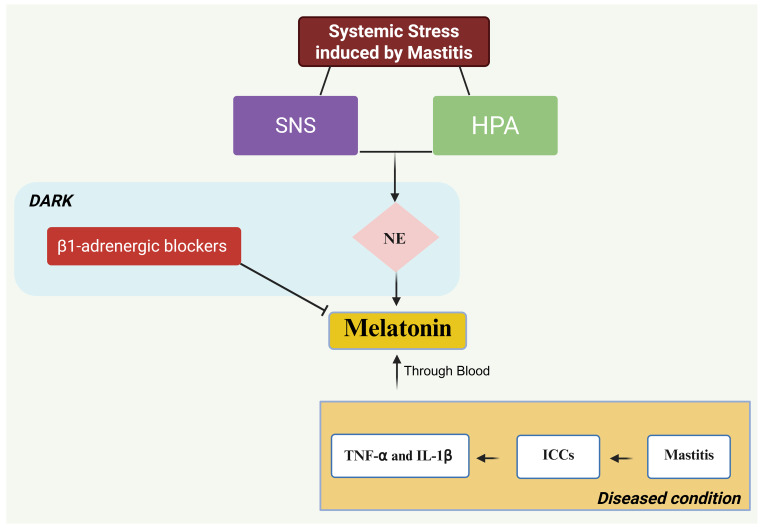
Secretion of melatonin during bacterial mastitis.

NE and IL-1 β are the two endogenous chemicals that regulate the secretion and function of melatonin (N-acetyl-5-methoxytryptamine). NE is under sympathetic nervous control (SNC) (Zefferino et al., 2021), while β 1-adrenergic blockers antagonize melatonin secretion in the dark (Cagnacci, 1996). According to the study by Pontes et al. (2006), it can be concluded that immunocompetent cells (ICCs) are the main source of melatonin production during mastitis, locally in the mammary gland tissue, in response to reactive oxygen species (ROS) generated during mastitis infection. Using *Escherichia coli* (in vitro), they observed an increase in melatonin produced by immunocompetent cells activated by *E. coli*. A significant increase in melatonin occurred at night in mothers with mastitis, which was correlated with TNF- α activation. The same results were also found by Shaheen et al. (2020) using milk samples. Some of the physical factors, like mastitis-associated pain and fever, which are two responsible components disrupting dark–light perception as well as sleep patterns, result in increased pineal gland secretion (Samanta, 2022; Carpenter et al., 2017). This is also evident in naturally occurring mastitis (clinical and subclinical), where TNF- α is one of the cytokines identified (Vitenberga-Verza et al., 2022). This means that melatonin secretion occurs in both types of mastitis because TNF- α and IL-1 β are the factors responsible for melatonin secretion (Pontes et al., 2006; Shaheen et al., 2020; Zefferino et al., 2021), as shown in Fig. 1.

### 4.1 Role of melatonin in mastitis

#### 4.1.1 Immunomodulatory effect of melatonin

**Figure 2:**
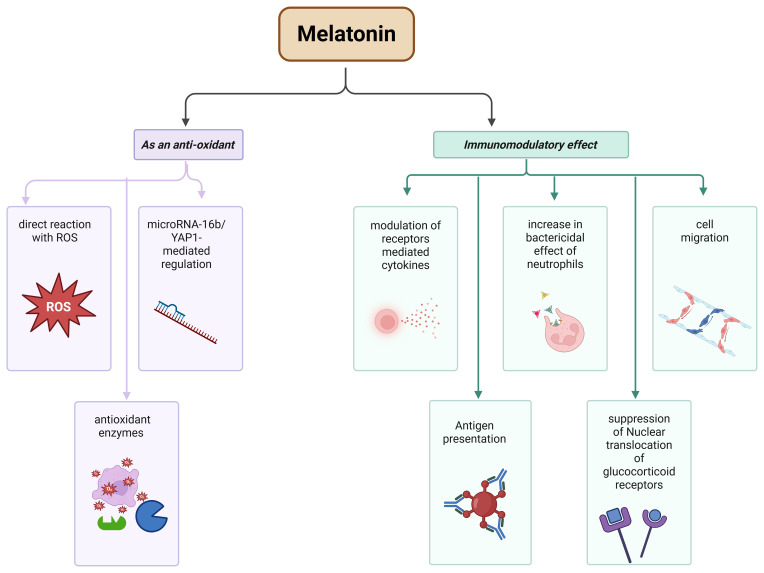
Role of melatonin during mastitis in the bovine mammary gland.

Melatonin has a key role in immune system modulation. In this context, melatonin, along with receptors, synchronizes the circadian rhythm through the suprachiasmatic nucleus (SCN) or directly by controlling the brain, thereby enhancing the immune response. This enhancement is achieved by modulating receptor-mediated cytokine production, pro-inflammatory cytokines (i.e., IL-6 and IL-8), cell migration, and antigen presentation to immunocompetent cells (Maestroni, 2024), as well as increasing the bactericidal effects of neutrophils (Xu et al., 2019). It also performs the suppression of nuclear translocation of glucocorticoid receptors (Maestroni, 2024) (see Fig. 2). These pathways need further investigation to know the molecular mechanism, such as exploring the interaction between melatonin and T-cells, because T-cells produce an important cytokine, i.e., IL-4, a promising cytokines during infection which could be estimated from their role of enhancing the proliferative effect of B-cells to Anti-IgM and as well as differentiation of T-helper 2 cells (Th-2 cells) (Keegan et al., 2021).

#### 4.1.2 As an antioxidant agent

In a diseased condition, “oxidative stress” is one of the major consequences of damaged cells or tissues. In oxidative stress, the production of reactive oxygen species (ROS) increases due to the inactivation of the antioxidant system, a well-known cause of inflammation and various serious diseases. It can lead to cancer, and cardiovascular, neurodegenerative, lung, renal, and other diseases. Oxidative stress is also a causative factor in aging (Reiter et al., 2016). During the inflammatory response, neutrophils, macrophages, and other immune cells produce ROS, along with other inflammatory mediators in response to mammary gland infection to combat infectious agents. But the increase in ROS also damages nearby tissues, leading to oxidative stress (Ryman et al., 2015).

Melatonin plays a key role in reducing oxidative stress by directly reacting with ROS, thereby acting as a free radical scavenger (Galano and Reiter, 2018). It is also now confirmed by the study of Chen et al. (2022b) that melatonin can reduce inflammation in the mammary gland (mastitis) via microRNA-16b/YAP1-mediated regulation. Some of the enzymes, such as superoxide dismutases, catalases, glutathione peroxidases, and glutathione reductases, are antioxidant enzymes that are activated by Metallothionein (MT1 and MT2) receptor signaling (Reiter et al., 2016; Hardeland and Pandi-Perumal, 2005; Ferlazzo et al., 2020). In mastitis (caused by *Staphylococcus aureus* bacteria), melatonin is also reported as an antioxidant and anti-inflammatory agent (Li and Sun, 2022) (see Fig. 2).

## 5 Impact of mastitis on milk production and composition (quality)

Somatic cell count (SCC) beyond 200 000 cells mL^−1^ is inversely proportional to yield, with a 2.5 % drop in production for every 100 000 cells mL^−1^ rise in SCC (Hisira et al., 2023). The advantages of keeping SCCs at around 90 000 cells mL^−1^ have been established by other researchers (Deluyker et al., 1993). In most developed farms worldwide, a minimum limit for SCCs in milk for human consumption is set, as shown in Table 2 (Sharma et al., 2011). The causative agents of mastitis vary in severity of pathogenesis. It is also clear that the various pathogens affect milk quality and composition differently, as shown in the study by Joy et al. (2023): *S. aureus* greatly affects milk production in dairy cows, and species belonging to the genus *Enterococcus* affect the somatic cell count (SSC), while *E. coli* has an insignificant effect on milk quality and composition. Apart from milk yield and composition, mastitis has a significant impact on cows' fertility (Schrick et al., 2001), which is also a challenge for researchers and warrants further investigation.

**Table 2:** This table shows various regulatory limits of milk SCCs for human consumption.

Country	Regulatory limits of (SCC)^*^	References
	cells mL^−1^	
European Union directives (92/46 CEE and 94/71 CEE)	400 000	Sharma et al. (2011);More (2009)
USA	750 000	
Much of Europe, New Zealand, and Australia	400 000	
Canada	500 000	
Brazil	500 000	Defante et al. (2019)
China	500 000	Han et al. (2020)
India	NA	Jadhav et al. (2016)
Switzerland	350 000	Kelly et al. (2011)
Sweden	400 000	More (2009)
Russia	700 000	Artem'eva et al. (2015)

^*^ These are the limits for buffalo milk. In cows' milk, SCC >200000 cells mL^−1^ indicates an unhealthy quarter, as discussed in the text. NA: in India, there are no such legal regulatory SCC standards for milk.

The signaling pathway involving cytokine-activated Janus kinase–signal transducer and activator of transcription (JAK-STAT) has an important effect on many physiological functions, including cell division, apoptosis, and mammary gland development. One example of this pathway is the way the hormone prolactin acts on it to regulate lactation (Khan et al., 2020). When mastitis occurs in a bovine mammary gland, the induction of Socs-3 in milk inhibits the JAK-STAT pathway, leading to decreased lactation (Zahoor et al., 2020). The most important causative factor for decreased quantity (especially quality) of dairy products is infection of the mammary gland by mastitis, which affects gene expression directly (i.e., at the level of mRNA) or indirectly (i.e., at the level of proteins produced). Kawecka-Grochocka et al. (2021) performed a study on the expression levels of alpha-S1-casein (CSN1S1) and kappa-casein (CSN3) in response to chronic mastitis (due to infection with either coagulase-positive staphylococci (CoPS) or coagulase-negative staphylococci (CoNS)) and bacteria-free (H) mammary tissue. They showed that the expression of both genes was similar across infections, but significant differences in protein production from those genes existed, suggesting that these differences are due to post-transcriptional and/or post-translational effects.

**Figure 3:**
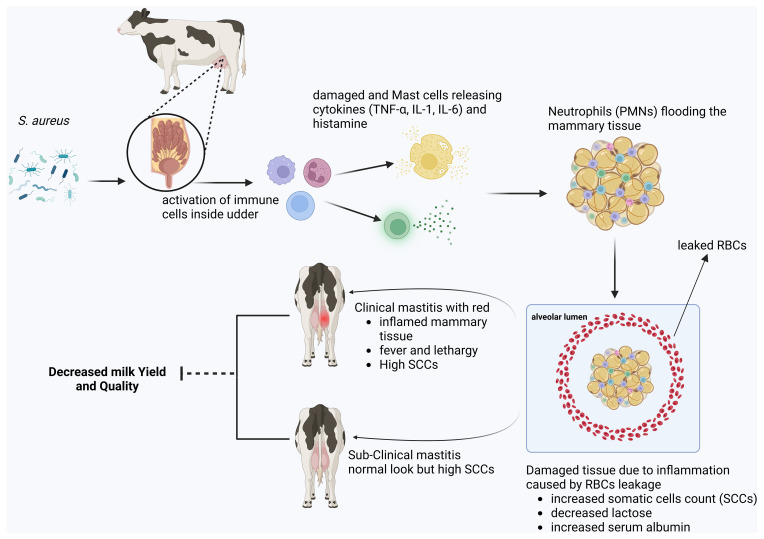
A schematic diagram illustrating the pathophysiology and economic impact of mastitis.

Milk quality or composition refers to the presence of various nutrients, such as proteins, fats, carbohydrates (lactose), SNFs, salt, and somatic cell count (SCC). It is now clear that milk production and composition are negatively correlated with mastitis (mammary gland inflammation). In this regard, the study conducted by Harjanti and Sambodho (2020) showed that, with increased mammary gland inflammation, milk production decreases, as do components such as proteins, fats, and carbohydrates, including lactose (see Fig. 3). The quality of milk is also correlated with the four seasons in the cows having subclinical mastitis, and we found that these four seasons have great impact upon the milk quality – not only the composition of the milk changes (Youssif et al., 2020) but also the various microbiota (i.e., bacteria, fungi, and other microorganisms) found in the milk showed a clear dynamics in prevalence (Antanaitis et al., 2021). However, recently, new research by Antanaitis et al. (2021) and Reitz et al. (2024) was conducted to diagnose subclinical mastitis at an early stage using milk lactose as a biomarker to improve animal health and milk quality.

## 6 Factors associated with severe mastitis

**Figure 4:**
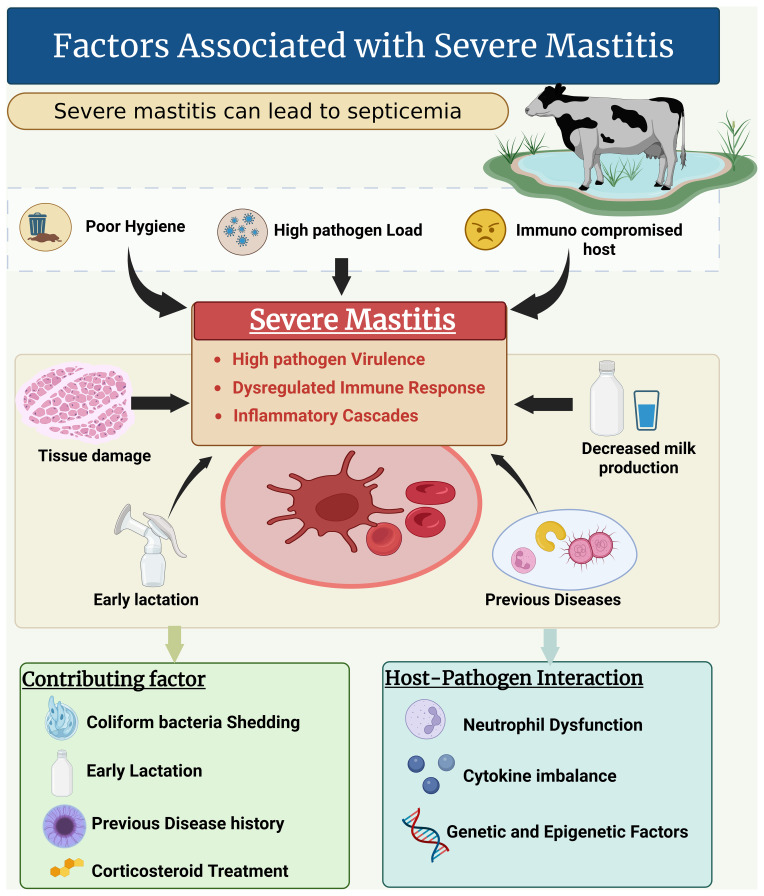
Factors associated with severe mastitis and its consequences.

The term “severe mastitis” means considerable disturbances posed by bacterial pathogens, which can lead to septicemia and even death of the cows. Common risk factors include poor hygiene, a high pathogen load, and an immunocompromised host (see Fig. 4). The severity of the infection depends primarily on the specific pathogen's virulence traits and host immune response variability. Mechanistically, severe mastitis is linked to heightened inflammatory cascades triggered by bacterial toxins and cell wall components, leading to extensive tissue damage, impaired milk production, and systemic symptoms (Sangiorgio et al., 2024). The host's innate and adaptive immune responses, including neutrophil function and cytokine profiles, critically determine the outcome, with dysregulated inflammation exacerbating severity. Many other factors investigated by Fredebeul-Krein et al. (2022) and Krebs et al. (2023) were associated with severe mastitis. These factors include (i) a combination and increase shedding of coliform pathogens, (ii) animal-related factors like stages of lactation (i.e., in early lactation, cows are more susceptible to severe mastitis), and a previous record of diseases prior to mastitis. Treatment with corticosteroids is also a major factor in severe disease progression in cows.

There is an urgent need for research to elucidate the molecular pathways mediating host–pathogen interactions that lead to severe inflammation and to identify biomarkers predictive of disease progression. Additionally, investigating genetic and epigenetic studies could further elucidate the factors influencing host susceptibility and immune regulation, offering personalized intervention strategies. The outcomes could also be increased by integrating environmental management with targeted antimicrobial and immunomodulatory therapies. Developing rapid diagnostic tools to differentiate severe from mild cases early could optimize treatment decisions and reduce complications.

## 7 Effects of mastitis on the expression of different genes

**Figure 5:**
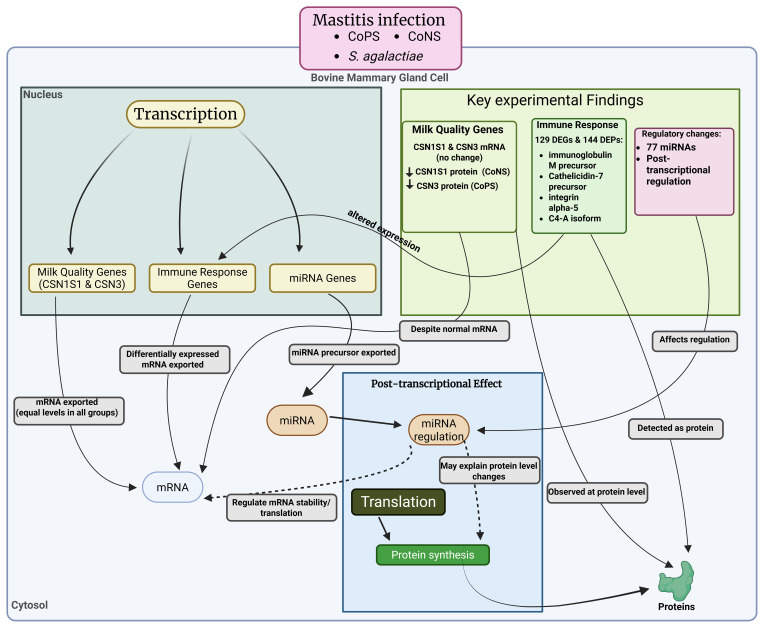
Effects of mastitis on CSN1S1 and CSN3 milk regulatory genes and proteins.

It was found that mammary gland inflammation greatly affects not only physical health but also the expression of genes related to milk quality, as revealed by Kawecka-Grochocka et al. (2021). They analyzed the expression of alpha-S1-casein (CSN1S1) and kappa-casein (CSN3) in three groups of cows, i.e., coagulase-positive staphylococci (CoPS), coagulase-negative staphylococci (CoNS), and a bacteria-free healthy group (H) (see Fig. 5). They found that the expression of these genes is the same across all groups, but there was a significant difference in protein levels: CoNS infection negatively affected the CSN1S1 protein level, and CoPS infection negatively affected the CSN3 protein level, suggesting that the effect is post-transcriptional. There are still many genes (Ma et al., 2021) involved in milk production and composition that need to be explored in response to mastitis.

Mammary gland inflammation also enhances host immune response by altering the expression and production of genes related to immunity. A study conducted by Zhang et al. (2018) explored how the host immune respond to mastitis induced by *S. agalactiae*. They found a total of 129 differentially expressed genes (DEGs, fold change >2 , p<0.05 ) and 144 differentially expressed proteins (DEPs, fold change >1.2 , p<0.05 ) in infected cows compared to a healthy group using microarray and isobaric tags for relative and absolute quantitation (iTRAQ). Among the genes and proteins, 18 DEGs/DEPs, immunoglobulin M precursor, cathelicidin-7 precursor, integrin alpha-5, and complement C4-A like isoform X1 were associated with *S. agalactiae*-induced mastitis. Moreover, mastitis was also co-related to microRNAs, and the study of Li et al. (2015) revealed a significant change in 77 miRNAs compared to control or healthy cows. Further investigation by KEGG (Kyoto Encyclopedia of Genes and Genomes) revealed that the associated genes are mostly related to endocytosis, and that olfactory transduction pathways involved cancer, and hence could be used for cancer therapy in future.

## 8 Economic and production impacts of mastitis

Mastitis is a serious biological problem because it is the least prevalent cause of mortality in milking cows (Ozbey et al., 2025). The study by Gantner et al. (2024) revealed that milk production decreases during mastitis, especially at its onset, as they observed the lowest milk yield on the day mastitis was detected. According to Esslemont and Kossaibati (1997), mastitis remains the most costly disease affecting dairy cattle, accounting for 38 % of total direct production costs. But nowadays, it is estimated that about 40 % of the 1.489 million cows worldwide suffer from mastitis (both types) (Zhylkaidar et al., 2021), which has caused about USD 147 per cow annually, as well as a loss of 11 %–18 % milk and the culling of production (Hogeveen et al., 2019). This increase posed a new challenge for researchers: why and how did it occur, and what strategies should be used to overcome it?

The economic importance of mastitis is prevalent worldwide (Zhylkaidar et al., 2021). In this section, we have tried to summarize the prevalence of mastitis in some of the Asian countries, such as South Asia and China. According to a report published by Chen et al. (2022a), the overall prevalence of bovine mastitis was 37.7 %, with the highest prevalence of 72 % in Inner Mongolia, as compared to the other sampled regions, which is 36.4 %–50.2 %. Similarly, in Pakistan, several studies indicate an average mastitis prevalence of 41.59 % (Khan et al., 2015; Ali et al., 2021; Shahzad et al., 2024; Haq et al., 2024). The prevalence of mastitis in India is also very high, i.e., the average prevalence from various studies is equal to 43.52 % (Kakati et al., 2024; Kumari et al., 2024; Rajkumar et al., 2024). Likewise, in Bangladesh, the prevalence is 55.63 % (Al Emon et al., 2024; Islam et al., 2025; Sajib et al., 2024), while in Nepal it is 23.7 % and in Türkiye it is 61.1 % (Chowdhury et al., 2024) (see Table 1). The overall prevalence of mastitis in some southern Asian countries, such as Sri Lanka and the Maldives, needs to be explored to determine the current status of mastitis and its risk factors, to inform the development of strategies (Pillai and Reji, 2023).

## 9 Diagnostic biomarkers

Diagnostic biomarkers are vital for both initial recognition and ongoing management of bovine mastitis. Table 3 lists diagnostic biomarkers associated with mastitis. One of the cheapest ways to diagnose mastitis and udder health is the somatic cell count (SCC) – studies have confirmed that in the presence of mastitis pathogens (clinical as well as in subclinical mastitis), the SCC is raised from the normal range (Tomanić et al., 2024a), i.e., 200 000 to 400 000 mL^−1^ (in subclinical) and >400000 (in clinical mastitis) (Tomanić et al., 2024a; Gantner et al., 2024). In recent studies, increasing concentrations of acute-phase proteins (APPs), such as haptoglobin and serum amyloid A, were observed during primary inflammatory responses as infections progressed throughout the mammary tissues (Huma et al., 2020). Enzymes such as lactate dehydrogenase and N-acetyl- β -D-glucosaminidase can indicate cellular damage after the onset of infection and the severity of the infection. Furthermore, there has been a growing emphasis on the use of molecular biomarkers (e.g., cytokines and microRNAs) as diagnostic markers of both subclinical and clinical cases of mastitis (Li et al., 2015). The development of advanced proteomic and metabolic profiling has enabled the identification of additional underutilized biomarker candidates, thereby supporting improvements in diagnostic precision and enabling more rapid therapeutic intervention. Collectively, these technologies enable more effective herd health monitoring and the opportunity to reduce financial losses associated with mastitis in cattle. It was also observed that there is a relatively low concentration of paraoxonase-1 (PON1) in the blood and milk of cows with subclinical mastitis (Nedić et al., 2019).

**Table 3:** Diagnostic biomarkers associated with mastitis.

S. No	Diagnostic biomarkers	References
1	>200000 SCCs mL^−1^ (subclinical)	Tomanić et al. (2024a);
	>400000 (clinical)	Gantner et al. (2024)
2	↑ Acute phase proteins (haptoglobin and serum amyloid-A)	Huma et al. (2020)
3	Lactate dehydrogenase and N-acetyl- β -D-glucosaminidase	Li et al. (2015)
4	Cytokines and microRNAs	
5	↓ Paraoxonase-1 (PON1)	Nedić et al. (2019)
6	WBCs and malondialdehyde (MDA)	Carvalho-Sombra et al. (2021);
		Huma et al. (2020)
7	Milk lactose	Antanaitis et al. (2021)

It is preliminarily important to develop cheap and convenient ways to diagnose mastitis, especially subclinical mastitis. Nowadays, various biomarkers have been identified, such as white blood cells (WBCs) and malondialdehyde (MDA) (Carvalho-Sombra et al., 2021; Huma et al., 2020), and milk lactose (Antanaitis et al., 2021), which make it easier to diagnose subclinical mastitis in early stages in bovine animals. Reductions in some antioxidant enzymes, such as glutathione peroxidase, superoxide dismutase, and catalase, are also important biomarkers in the diagnosis of early mastitis in bovines (Huma et al., 2020).

## 10 Treatment and control of mastitis

### 10.1 Control

One of the main reasons that mastitis cannot be eradicated is that it is multifactorial; however, it can be minimized using systematic control and prevention programs. To effectively control and prevent mastitis, there must be an integrated herd management program that incorporates animal health, hygiene, nutrition, housing, milking practices, and specific treatment protocols. The two primary objectives for controlling mastitis are (1) to shorten the time frame of existing intramammary infections and (2) to reduce the frequency of new intramammary infections. Mastitis is caused by the interaction of pathogens, host immunity, and environmental factors; if mastitis is to be successfully controlled, there must be a uniform implementation of a broad range of preventative measures at the farm level (Zigo et al., 2021; Benić et al., 2018).

One of the key tools in mastitis control is the somatic cell count (SCC). SCC is the primary means of monitoring udder health and is composed primarily of white blood cells that increase in response to an inflammatory response in the udder. An increase in the somatic cell count indicates that a cow has subclinical or clinical mastitis, and will have lower milk production and quality than those with lower SCC. Bulk tank SCC is used as a measure of the udder health of the entire herd; when bulk tank SCC exceeds threshold limits (for example, 400 000 cells mL^−1^), corrective actions are needed (Zajác et al., 2012). The management options associated with high SCC include identifying and segregating cows with a high somatic cell count; cows with a high SCC should always be milked last in order not to contaminate the bulk tank with these cows' milk. Chronically infected cows should be removed, especially those with recurrent infections due to contagious organisms such as *Staphylococcus aureus*. Early diagnosis should be done using tests such as the California mastitis test (CMT) and the culture of bacteria (Sharma and Jeong, 2013).

Rapidly cultured milk samples provide the opportunity to differentiate between Gram-positive and Gram-negative bacteria, and will assist in selecting appropriate antibiotics to treat the identified pathogens. Treatment for identified pathogens typically comprises first-, second-, and third-line antibiotics, depending on the pathogen's susceptibility to each class. Treating the infection early with the appropriate antibiotic will improve recovery rates, reduce SCC, and decrease the likelihood of antibiotic resistance developing (Reksen et al., 2006).

For the mammary gland to maintain its immune competence, there must be a balanced approach to nutrition where deficiencies or imbalances will result in a weakened ability of leukocytes (white blood cells) to function properly, decreased ability of antibodies to be transported to the mammary gland, and damage to the tissue surrounding the glands, all of which contribute to an increased risk of infection. A feeding program must therefore take into account the following: an appropriate/equilibrated energy and protein intake, sufficient levels of vitamins and minerals, and protection against the ingestion of mycotoxin-contaminated feed. Deficiencies in vitamins and minerals can have an indirect effect on the increase of mastitis through a reduction in the overall function of the immune system and a delay in closure of the teat canal after milking due to metabolic disorders (hypocalcemia, ketosis, and fatty liver disease) that arise from poor nutrition (Zigo et al., 2014; Nickerson et al., 2013; Eastridge, 2006; Cobirka et al., 2020).

The role of vitamin E and selenium (Se) as important antioxidants protecting mammary cells from oxidative damage and enhancing neutrophil (white blood cells) bactericidal activity is well established. When animals have low levels of either of these vitamins, they will likely have a higher incidence and severity of clinical mastitis, more retained placentas, and an impaired immune system. Supplementation of vitamin E and selenium, either alone or in combination, will produce many positive outcomes, including decreased somatic cell counts (SCCs), reduced length of clinical mastitis, increased quality of milk produced, and increased resistance to infectious agents like *E. coli* and *S. aureus*. There is an additive/synergistic effect when both vitamin E and selenium are supplemented together; they are especially important during the dry and early lactation periods (Zigo et al., 2014; Oltramari et al., 2014).

Humic acids are new feed additives used as an alternative to antibiotics for mastitis prevention. Their potential benefits include binding toxins, stabilizing rumen microflora, stimulating the immune response, suppressing the growth of pathogenic microorganisms, and decreasing SCC and the incidence of subclinical mastitis. Humic acids will aid in developing sustainable means for controlling mastitis under guidelines restricting the use of prophylactic antibiotics (Mudroňová et al., 2020; Semjon et al., 2020).

#### 10.1.1 Housing management and herd environment

Environmental hygiene is paramount in controlling pathogens that lead to environmental mastitis. Providing adequate housing will ensure a clean, dry, and comfortable environment for the cattle of the farm; this in turn will reduce the possibility of bacteria being in contact with teat and udder. The following practices should be followed (Haley et al., 2001; Broucek et al., 2015; Leach et al., 2015):

- Regular removal of manure;
- Frequent replacement of bedding material (and disinfection of bedding);
- Use of lime or commercial drying agents;
- Adequate ventilation and stall design.

The use of recycled solid manure (if recycled solid manure is used as bedding) must be processed through some type of treatment to guarantee proper hygiene or cleanliness. Improvements in the bedding composition have been demonstrated to decrease both the number of bacteria residing in the bedding and the incidence of clinical mastitis in a herd. Contagious pathogens are also isolated when separating sick cows from healthy cows, and providing a grouping system for first-calf heifers, thus lessening the transmission of contagious pathogens (Popescu et al., 2014; Fournel et al., 2019).

#### 10.1.2 Lactation management and hygiene in milk production

The method used for milking has a direct bearing on the incidence of mastitis. Following milking, the teat canal will stay open for approximately 2 h, allowing microbial organisms to enter the udder through the open teat channel if hygiene conditions are inadequate. Control measures should be implemented to monitor udder health and include routine CMT testing of cows shortly after calving, the early culturing of cows with a positive CMT test, milking infected cows last, the routine disinfection of teats and udder prior to and following milking, and the routine cleaning and sanitation of milking equipment and milking facilities (Jackson et al., 2002). In many cases, subclinical mastitis is not diagnosed and goes untreated, therefore routine monitoring and making treatment decisions based on the bacteriological testing of the milk from cows with subclinical mastitis is essential. Milk safety more than just protects the udder of the dairy animal; it also keeps the end product safe for human consumption (Tančin et al., 2007; Hovinen and Pyörälä, 2011).

#### 10.1.3 Drying cow

The period in which a dairy cow is not producing milk (the dry period) represents an important stage in the management of dairy cows since it provides an opportunity for the mammary gland tissue to regenerate and recover from the previous lactation and for the udder to prepare for the upcoming lactation. Proper management during this time has significant physiological, morphological, and immunological consequences for maintaining udder health and preventing mastitis (Berry et al., 2004; Berry and Hillerton, 2002).

In nature, cows have a natural means of protecting themselves against intramammary infection during the dry period through the formation of a keratin plug in the teat canal. This keratin plug serves as a physical barrier that prevents pathogenic bacteria from entering the udder. However, the amount of time it takes for the teat canal to completely close after drying off varies between cows. Research has shown that approximately 50 % of teats close within the first week after drying off, whereas some teats will remain open for weeks and others may never close completely. Teats that do not form a proper keratin seal are at greater risk of developing new intramammary infections (Williamson et al., 1995).

If a cow develops an infection in any of the quarters of her udder during the dry period or has a quarter that is still infected from the previous lactation, she will experience a significant decrease in milk yield during the next lactation (often a 30 %–40 % decrease) (Nickerson and Ryman, 2019). The risk of developing a new intramammary infection is highest at the beginning and at the end of the dry period. Therefore, it is critical to apply strict sanitation and proper preventative treatments during the dry period.

Cows should be dried off approximately 60 d before they are due to calve. If a cow is producing over 25 L of milk per day, she may have an approximately 10 d reduction in the amount of time that she is dried off before she calves (Kaczorek-Łukowska et al., 2021). Cows should be evaluated for mastitis using the California mastitis test (CMT) prior to drying them off. If a cow is detected as having mastitis, that cow should receive treatment prior to being dried off. Traditionally, blanket dry cow therapy, where all quarters of all cows receive long-acting intramammary antibiotics at drying off, is one of the most effective methods of controlling mastitis. This method reduces the risk of existing infections (mainly caused by contagious pathogens) and the risk of developing new intramammary infections (primarily caused by environmental pathogens). The combined use of intramammary antibiotics and internal teat sealants (combo therapy) provides better protection against mastitis than each product used alone because both cure existing infections and form a physical barrier in the teat canal against future infections (Tiwari et al., 2013; White et al., 2011; Sol et al., 1994; Cvetnić et al., 2016; Cvetnić et al., 2021).

Due to increasing legislative and consumer pressure to reduce antibiotic use, many producers are now using selective dry cow therapy. Under selective dry cow therapy, producers have classified cows into three groups based on their somatic cell count (SCC) history, performance during clinical examination, and mastitis record. These groups are shown below (Crispie et al., 2004; Kaczorek-Łukowska et al., 2021; Zigo et al., 2021; Hassan et al., 2020):

1. Low-risk cows ( <200000 SCC mL^−1^ and no mastitis history) will not be treated with antibiotics but will be treated with only a teat sealant at drying off.
2. Moderate-risk cows (elevated SCC or previous mastitis without current clinical signs) will be treated with a long-acting intramammary antibiotic and a teat sealant at drying off.
3. High-risk cows (cows with current clinical or chronic mastitis) will be treated with antibiotics and a teat sealant after being treated with antibiotics and a teat sealant during lactation.

This targeted approach protects against the unnecessary use of antibiotics while still maintaining the health of the udder. The use of probiotics as adjunctive or alternative means of restoring the healthy intramammary microbiota during the dry period has been examined. The application of certain Lactobacillus species to the udder has been shown to have limited efficacy against minor pathogens (i.e., coagulase-negative staphylococcus), but no demonstrated efficacy against all major mastitis pathogens (i.e., *Staphylococcus aureus, E. coli*). Therefore, probiotics will be considered an adjunct as opposed to the primary treatment.

### 10.2 Treatment and prevention

The effect of homeopathic treatment has been studied by researchers to treat bacterial infections from the genus *Staphylococcus (S. aureus* and *S. epidermidis)*. The similarity of the gene *icaADBC* and their expression related to the production of biofilm confirmed that homeopathy is not an effective treatment for mastitis in animals and needs some strategies to control the disease (Ferreira et al., 2022). Antibiotics is the most effective way of controlling mastitis and is widely used across the world (Li et al., 2023). Apart from the significance of antibiotics, it is also observed that the use of antibiotics has a great impact on the animals’ health because of the adaptive nature of the bacteria providing resistance to these antibiotics (Awandkar et al., 2022), which would pose a more severe risk to the animals’ health in future (see Fig. 3). A challenge for researchers is to explore the resistance posed by bacteria to antibiotics.

Recently, a therapeutic strategy was developed by Ranjani et al. (2022) in the treatment of the strains of different mastitis-causing bacteria (i.e., *Acinetobacter junii*, *Klebsiella pneumoniae*, *Pseudomonas stutzeri*, and *Acinetobacter baumannii*) with the extracts of *Syzygium aromaticum*, *Cinnamomum verum*, *Emblica ofcinalis*, *Terminalia belerica*, *Terminalia chebula*, and *Cymbopogon citratus* (a polyherbal nanocolloid, or PHNc). The study revealed that PHNc can be used as bacteriostatic, bactericidal, and antibiofilm activity against all the strains; consequently, it reduces the growth of mastitis-causing pathogens. Similarly, neem (*Azadirachta indica*) was also proved to have a bacteriostatic effect (Karunathilaka et al., 2024). Nowadays, in this post-antibiotic era, researchers have developed another significant and novel strategy to control mastitis, as reviewed by Nale and McEwan (2023) and Touza-Otero et al. (2024): the bacteriophage virus was used for the control of different mastitis-causing bacteria by taking advantage of the phage specificity and other characteristics, such as the lysis of the bacterial membrane, adsorption, and penetration power (see Fig. 7).

**Figure 6:**
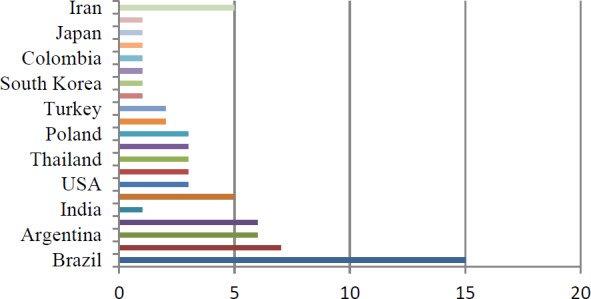
Proportion of conducted studies on the treatment of mastitis by essential oils (adopted from Kovacevic et al., 2025).

**Figure 7:**
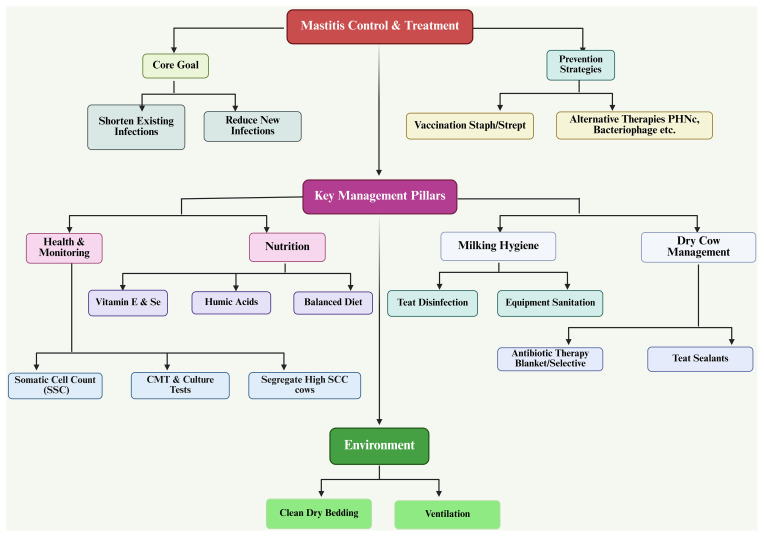
Different strategies used to control and treat mastitis.

As natural alternatives to conventional antibiotics in veterinary medicine with low cost (Kovačević et al., 2022a; Tomanić et al., 2024b), essential oils are increasingly being recognized as a way to combat global antimicrobial resistance. Essential oils are the extract obtained from plants like *Thymus vulgaris* (thyme) and *Origanum vulgare* (oregano), which exhibit antibacterial activity against mastitis pathogens by disrupting their membranes and biofilms. Many of the essential oils have antimicrobial, antioxidant, anti-inflammatory and/or immune enhancement properties; many are considered safe for use. In dairy herds, essential oils are administered via four main methods – intramammary infusion, topical application, oral supplementation, and environmental disinfection – as ways to treat or prevent infections such as mastitis.

The mechanisms by which essential oils exert their antimicrobial influence against pathogens associated with mastitis are diverse. The most common action by which essential oils exert their antimicrobial effect against pathogenic bacteria is by disrupting the membrane of the bacterial cell. This disruption of the integrity of the bacterial cell membrane results in the loss of an ion gradient, leading to the lysis of the bacterial cell. These components (thymol, carvacrol, and eugenol) penetrate the membrane of the bacterial cell, adversely affecting the activity of essential functions (e.g., electron transport, protein synthesis, and enzyme activity, such as ATPase) of the bacterial cell. Essential oils can leak nicotinamide adenine dinucleotide + hydrogen (NADH) from the bacterial cell, thereby damaging the metabolism of the bacterial cell; essential oils can also interact with DNA in resistant bacteria such as methicillin-resistant *Staphylococcus aureus* (MRSA) to decrease toxin production in the bacterial cell. The bacteriostatic and/or bactericidal effect of each essential oil varies based upon concentration. The presence of synergistic interactions among the components of each essential oil will increase the efficacy of the essential oils, whereas the presence of antagonistic interactions among the components of each essential oil will compromise the bioactive effects of the essential oils. Current barriers to the acceptance of these products by the veterinary community include (1) variability in chemical composition, (2) the absence of standardized dosages or testing protocols, and (3) very few clinical studies of essential oils done in vivo. Future integration of essential oils into veterinary practice will depend on standardized preparations, successful clinical trials, and effective delivery systems to ensure efficacy and safety. Furthermore, Fig. 6 shows recent studies conducted in this area of interest from various regions of the world. However, some countries still lack these types of studies, which means that these countries need to focus on treatment like this, which is beyond antibiotics and antimicrobial resistance (Kovacevic et al., 2025).

#### 10.2.1 Vaccination procedures against mastitis pathogens

Vaccination as a strategy for preventing intramammary infections in dairy cows is an immunoprophylactic approach that increases dairy cows' specific immunity against the organisms that cause these infections. Although vaccination does not completely prevent mastitis, when used in conjunction with other control programs, it can reduce the severity, duration, and economic impact of mastitis (Boerhout et al., 2015).

Mastitis vaccines generally work by increasing the concentration of specific antibodies in the blood, which can then be transferred to milk. However, the transfer of antibodies from blood to milk does not occur by passive diffusion. Instead, it is an active transport mechanism that depends on the physiological state of the mammary gland. This limits the effectiveness of vaccines, especially when mammary gland function is compromised (Zigo et al., 2021).

In addition, another limitation of vaccination is the reduced immunological function of immune mechanisms in milk. There are lower concentrations of complement proteins in the milk than in blood, and the neutrophils of milk are less functionally active than those found in blood. The bactericidal activity of neutrophils is limited by the low levels of oxygen and glucose in milk, and the presence of fat globules can interfere with the ability of neutrophils to phagocytose an organism. These limitations reduce the ability of the immune cells of milk to kill pathogens in the mammary gland, even in vaccinated cows (Ulfman et al., 2018).

Currently available vaccines for mastitis are either monovalent (for the prevention of *Staphylococcus aureus*) or polyvalent (for the prevention of *Staphylococci* and *Streptococci*). Monovalent and polyvalent vaccines will not necessarily prevent either clinical or subclinical mastitis, but they can reduce the duration of disease, reduce levels of bacterial shedding, and reduce the severity of the clinical signs. Vaccination resulted in a reduction of somatic cell counts (SCCs) and a reduction in the incidence of clinical mastitis in multiple studies, especially in herds with chronic problems with mastitis. Polyvalent vaccines have been recommended for herds that experience chronic mastitis problems as a result of coliform bacteria, *S. aureus*, and coagulase-negative staphylococci. Vaccinated cows had lower SCC levels and a lower incidence of mastitis than unvaccinated herd mates. In some mastitis vaccination programs, treated herds reported that chronic mastitis incidents were reduced by up to 40 % through the use of vaccination (Zigo et al., 2021; Toušová et al., 2011).

The standard schedule of vaccination for mastitis is three doses: the first dose is given at the time of drying off, the second dose at 1 month after the first dose, and the third dose approximately 2 weeks after the birth of the calf. The greatest efficiency of vaccination has been demonstrated in cows that have had at least one previous lactation, with an approximate 10 %–20 % protection in decreasing both clinical and subclinical mastitis. For the best protection in the long term, it is recommended that heifers receive their initial doses of vaccines prior to their first breeding, and that booster doses are administered at 6-month intervals and at the time of calving (Doležal and Kopunecz, 2010).

While vaccination has some advantages and benefits, vaccination is financially costly and will not be a substitute for basic preventive principles of mastitis control, including good hygiene, proper nutrition, the monitoring of SCC levels, and the use of effective milking management techniques; therefore, vaccination should be viewed only as a complementary tool, and not as a substitute, to the basic principles of herd management. When vaccination is used in conjunction with good herd management practices, it can improve udder health, decrease SCC levels, reduce the severity of clinically diagnosed mastitis cases, and ultimately increase the quality of milk produced and the profits for the dairy industry (Zigo et al., 2021).

#### 10.2.2 Nanotherapy

The growing field of nanotechnology is providing new tools that will continue to shape the future of veterinary medicine in the 21st century, particularly in disease prevention, diagnosis, and treatment. The use of nanotechnology will also provide alternative means of treating diseases, such as bovine mastitis, by reducing reliance on traditional antibiotics, thereby limiting the development of antimicrobial resistance (AMR) and limiting drug residues in food products. Additionally, nanoparticles serve as effective drug-delivery vehicles that can target affected cells, enabling lower dosages, shorter withdrawal times, lower overall costs, and fewer side effects. Nanoparticles offer biopharmaceutical advantages, including increased intracellular drug uptake, extended drug retention, and antibiofilm activity. There is evidence that specific types of nanoparticles, such as silver, copper, zinc oxide, chitosan, and nanogel, have demonstrated effectiveness against the pathogens that cause mastitis, including antibiotic-resistant *Staphylococcus aureus*. The use of synergistic combinations of nanoparticles (e.g., silver and antibiotics) has the potential to further enhance their effectiveness in combating mastitis. As a result, veterinary nanomedicine was developed as an innovative approach to enhance the health and welfare of the world's livestock, thereby increasing production in the livestock industry (Tomanić et al., 2023a).

## 11 Antimicrobial resistance (AMR)

While treating mastitis, the most common approach is the use of antibiotics (Tomanić et al., 2023b), which pose a significant challenge for researchers due to bacterial resistance to these antibiotics (Awandkar et al., 2022). To assess the resistance of a pathogen, especially of bacteria, many studies have been conducted to evaluate the pathogens' responses to administered antibiotics. Table 4 summarizes the major mastitis-causing bacteria along with their mono and multidrug resistance. Researchers are working to combat this biological problem from different perspective, such as the study conducted by Kovačević et al. (2022b) to correlate antimicrobial use (AMU) with antimicrobial resistance (AMR), confirming a pattern that is a positive sign for good veterinary and clinical practice in combating the threat of AMR globally.

**Table 4:** Bacterial strains and their resistance to antibiotics.

Bacteria	Resistant to	References
*Staphylococcus aureus*	Penicillin, clindamycin,erythromycin, gentamycin, ceftiofur,cephalothin, nitrofurantoin,sulfamethoxazole/trimethoprim,ceftriaxone	Pitkälä et al. (2004);Molineri et al. (2021);Zhang et al. (2022);Kovačević et al. (2022b)
*Streptococcus uberis*	Oxacillin, gentamycin, tetracycline,	Boireau et al. (2018)
*E. coli*	Cephalosporin, amoxicillin,tetracycline, ceftriaxone	Boireau et al. (2018);Kovačević et al. (2022b)
Coagulase-positive staphylococci (CoPS)	Penicillin, Amoxicillin, tetracycline	Boireau et al. (2018)
Coagulase-negative staphylococci (CoNS)	Macrolide	Zigo et al. (2022)
*Mycobacterium* spp.	Clarithromycin	Cvetnić et al. (2022)

## 12 Comparative strengths and limitations of control approaches

This article summarizes existing knowledge of bovine mastitis; indeed, mastitis is both an ongoing source of economic burden for the dairy industry and an ongoing animal welfare concern. Additionally, there are emerging threats posed by the issue of increased levels of antimicrobial resistance (AMR). A thorough evaluation of the causes, diagnosis, effects, and control measures associated with bovine mastitis has produced numerous important findings as well as implications for future research.

The use of traditional antibiotics is the most effective and fastest method of treating clinical mastitis; however, the increasing level of AMR is threatening the effectiveness of these drugs and raising consumer concerns about their use as a food safety hazard. Conversely, various preventive approaches can be used to achieve sustained reductions in the incidence of mastitis, such as improving milking hygiene practices, dry cow management (including selective dry cow therapy), and nutritional supplements such as vitamin E and selenium. However, these require continual long-term herd management commitment and cannot be used to cure acute cases of mastitis. Emerging control alternatives, including the use of natural plant extracts (essential oils), nanotechnology, and bacteriophages, have demonstrated promising in vitro effectiveness and a low risk of developing AMR; however, these alternatives are currently limited by a lack of standardized products, the high cost, and limited in vivo evidence from large trials. Vaccination is also a proactive method of providing a robust immunoprotective response to certain mastitis pathogens, most notably *Staphylococcus aureus*; however, vaccination does not replace good hygiene practices. The use of endogenous factors like different gene expressions helps in angiogenesis and cell proliferation. For example, from the study of Zhang et al. (2025), mRNA therapy offers a safer and more scalable alternative. They demonstrated that LNP@VEGF effectively protected mRNA from degradation and enabled robust vascular endothelial growth factor-A_165_ (VEGF-A) expression in vitro, promoting endothelial proliferation. This therapy could also enhance the treatment strategy for bovine mammary epithelial cells (BMECs).

### 12.1 Practical aspects and on-farm feasibility

It is critical that the economic realities of dairy farming be incorporated into control strategies, recognizing the need for cost vs. efficacy and practicality. Despite their advantages in providing early and accurate diagnosis through advanced diagnostics such as molecular biomarkers and proteomics, their high cost makes them more suitable for large-scale or high-value dairy farms. Conversely, the primary on-farm monitoring techniques available to the producer for udder health are the somatic cell count (SCC) and the California mastitis test (CMT) because of their ease of use and low cost, even though they do not provide a direct measure of udder health. A successful example of how strategy can improve the feasibility of dry cow therapy is the transition from blanket dry cow therapy to selective dry cow therapy, which reduces antibiotic use while maintaining cost-effectiveness and udder health. The successful implementation of selective dry cow therapy will require accurate recordkeeping and diagnostic expertise.

### 12.2 Synergy and combination strategies

It has been emphasized that multifaceted, integrated approaches are fundamental to mastitis control; therefore, no single strategy alone will be adequate. Combining prevention (hygiene, vaccination, nutrition), monitoring (SCC, targeted diagnostics), and therapeutic intervention can be used to achieve synergy; for example, the combined use of internal teat sealants with antibiotics at drying off provides synergistic protection. Future strategies may specifically incorporate natural antimicrobials (essential oils) and nanotechnology to enhance delivery and treatment effectiveness, or combine phage therapy with immunomodulators (melatonin) to target both pathogens and to reduce inflammation.

### 12.3 Research gaps and future directions

Although great progress has been made, there are still many large gaps. To begin with, we do not understand much about the genetic basis of each cattle breed's susceptibility to disease, making it difficult to use this knowledge for breeding programs to breed resiliency into future generations. Additionally, we know little about how the mammary and gut microbiomes affect infection dynamics and recovery; this represents an exciting area that may provide a pathway for probiotics/prebiotics. Furthermore, molecules such as melatonin show immunomodulatory effects, although their exact molecular mechanisms and therapeutic potential require extensive in vivo studies. There is also a critical need for standardized, well-controlled clinical trials of novel therapies (such as phage, nanoparticles, or herbal composites) to establish valid evidence of their efficacy, dosing, and safety for animal use.

## 13 Conclusion

The conclusion of this review affirms that bovine mastitis remains an economically important and serious disease caused primarily by bacterial pathogens, particularly *Staphylococcus* spp. The development of diagnostic advances, such as biomarkers and the determination of somatic cell counts (SCCs), will allow for earlier identification of bovine mastitis; however, the emergence of antimicrobial resistance (AMR) will present an additional challenge regarding treatment options (e.g., antibiotics). Examples of novel treatments and supplementation could include improved herd management practices, improved nutrition, vaccination, and the use of new techniques (including phage therapy, essential oil therapy, and nanotechnology). Future research efforts should focus on developing a better understanding of the mechanisms of genetic resistance to mastitis in cattle; the effect of the mammary microbiome and gut microbiome on the development of bovine mastitis; and the potential of immunomodulatory agents (e.g., melatonin) to develop sustainable, integrated control programs for controlling bovine mastitis.

## Data Availability

All the data generated or analyzed during this study are included in this published article.

## Appendix A Abbreviations

**Table:**

**Abbreviations**	**Meaning**
SCC	Somatic cell count
HPA	Hypothalamic-pituitary-adrenocortical axis
SN	Sympathetic nervous system
IFN	Interferon
SNC	Sympathetic nervous control
ICCs	Immuno competent cells
NE	Norepinephrine
SCN	Suprachiasmatic nucleus
ROS	Reactive oxygen species
MT	Metallothionein
AK-STAT	Janus kinase–signal transducer and activator of transcription
CSN1S1	Alpha-S1-casein
CSN3	Kappa-casein-3
CoNS	Coagulase-negative staphylococci
CoPS	Coagulase-positive staphylococci
SNFs	Solid not fats
DIF	Difenoconazole
ER	Endoplasmic reticulum
MMP	Mitochondrial membrane potential
DEGs	Differentially expressed genes
DEPs	Differentially expressed proteins
KEGG	Kyoto Encyclopedia of Genes and Genomes
MDA	Melondialdehyde
PHNc	Polyherbal nanocolloids

## Code availability

No software and/or code were used while writing this narrative review.
